# Hsa-miR-429 promotes bladder cancer cell proliferation via inhibiting CDKN2B

**DOI:** 10.18632/oncotarget.19878

**Published:** 2017-08-03

**Authors:** Jiangeng Yang, Yuchen Liu, Anbang He, Yuhan Liu, Jianting Wu, Xinhui Liao, Zhaojie Lv, Feng Wang, Hongbing Mei

**Affiliations:** ^1^ Department of Urology, Shenzhen Second People’s Hospital, Clinical Institute of Guangzhou Medical University, Shenzhen 518039, Guangdong Province, China; ^2^ Key Laboratory of Medical Reprogramming Technology, Shenzhen Second People’s Hospital, The First Affiliated Hospital of Shenzhen University Shenzhen, Guangdong Province, China; ^3^ Anhui Medical University, Anhui, China

**Keywords:** microRNA, urothelial carcinoma, cyclin-dependent kinase inhibitor 2B

## Abstract

**Background and Objectives:**

Hsa-miR-429 is increased in bladder cancer. Its roles in bladder cancer are poorly understood.

**Methods:**

The expression levels of hsa-miR-429 and cyclin-dependent kinase inhibitor 2B (CDKN2B) were determined using Real-Time qPCR in a total of 50 patients with bladder cancer. Bladder cancer T24 and 5637 cells were transfected CDKN2B siRNA or hsa-miR-429 mimic. CDKN2B expression levels after transfection were detected by Real-Time qPCR and Western blot assay respectively. Binding sites between hsa-miR-429 and 3’-untranslated region of CDKN2B were confirmed by Dual luciferase reporter assay. Cell proliferation was evaluated using MTT and EdU assays. Cell apoptosis was determined using ELISA assay.

**Results:**

Higher hsa-miR-429 expression levels were associated with higher tumor grade and stage. All patients with low hsa-miR-429 expression survived 5 years, while with high hsa-miR-429 expression, only 58% survived. Hsa-miR-429 and CDKN2B were inversely expressed in bladder cancer. Hsa-miR-429 mimic decreased the expression of CDKN2B at both mRNA and protein levels. The binding site was confirmed between hsa-miR-429 and 3’-untranslated region of CDKN2B. Up-regulation of hsa-miR-429 or down-regulation of CDKN2B promoted cell growth and decreased apoptosis.

**Conclusions:**

Our data suggest a mechanism for hsa-miR-429 to play oncogenic roles via inhibiting CDKN2B.

## INTRODUCTION

About 72,500 cases of newly diagnosed bladder cancer and 17,960 bladder cancer deaths are estimated to occur in the United States in 2013 [1]. Urothelial carcinomas comprise around 90% of bladder cancers. However, the carcinogenesis of urothelial carcinoma is poorly perceived [2].

The complex phenotypes of bladder cancer cells are controlled by functional response genes [3]. MicroRNAs are small non-coding RNAs that mainly inhibit gene expression [4]. They are increased or decreased in cancers and able to promote or suppress cancer development [5]. Hsa-miR-429 is increased in cancers, such as endometrial carcinoma [6] and colorectal cancer [7]. In contrast, some other findings suggest that hsa-miR-429 functions as a tumor suppressor in osteosarcoma [8], renal cell carcinoma [9] and cervical cancer [10]. We have validated that hsa-miR-429 is indeed increased in bladder cancer [11], while it has been found that hsa-miR-429 reverses epithelial-mesenchymal transition by restoring E-cadherin expression in bladder cancer [12]. Hsa-miR-429 belongs to miR-200 family, and the upregulation of miR200 family has also been reported in bladder cancer [13]. For example, there should be a positive relationship between high microRNA-200C expression and the risk of death from disease in muscle-invasive urothelial carcinoma of the bladder, as revealed by one published work [14].

Partial sequence pairing between miRNA and target mRNA leads to translation inhibition and/or mRNA degradation in animals [15]. We have identified that hsa-miR-125b decreased its target SIRT7 at both mRNA and protein levels through partial sequence pairing with the target sites [16]. Cyclin-dependent kinase inhibitor 2B (CDKN2B) is a tumor suppressor [17]. Deletion, mutation and hypermethylation of CDKN2B gene have been reported to lead to the loss of CDKN2B expression [18]. There is a negative regulatory role of miR-15a-5p in the apoptosis of smooth muscle cells via binding to the CDKN2B mRNA 3’UTR [19]. *In silico* analysis indicates that CDKN2B 3’UTR has binding site for hsa-miR-429. This finding proposed an important question about the relationship between hsa-miR-429 and CDKN2B in bladder cancer. We hypothesized that hsa-miR-429 may play oncogenic roles via inhibiting CDKN2B in bladder cancer.

To test the proposed hypothesis, in this study, the expression patterns of hsa-miR-429 and CDKN2B in urothelial carcinoma of the bladder were determined. Hsa-miR-429 mimic decreased CDKN2B at both mRNA and protein levels. The binding site between hsa-miR-429 and CDKN2B 3’UTR was confirmed. We found that hsa-miR-429 promoted cell growth and decreased apoptosis via inhibiting CDKN2B.

## RESULTS

### Hsa-miR-429 and CDKN2B were inversely expressed in bladder cancer

**Figure 1:**
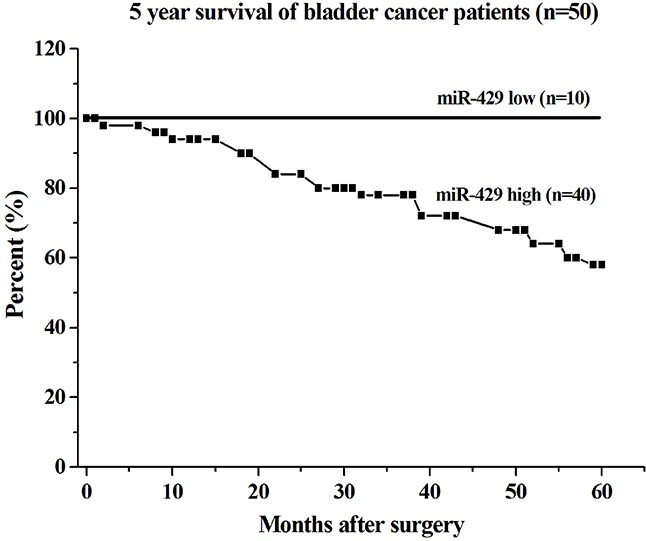
Correlation of hsa-miR-429 expression with 5 year survival of bladder cancer patients The survival rate of the group with low expression of miR-429 was lower than that of the group with high expression of miR-429 (log-rank test, p < 0.01).

The relative expression levels of Hsa-miR-429 and CDKN2B were evaluated using Real-Time qPCR in 50 patients with bladder cancer. Hsa-miR-429 was increased in bladder cancer compared with matched normal urothelium. As shown in Table 1, upregulation of hsa-miR-429 was positively correlated with bladder cancer clinical pathologic grading (*p*<0.001) and TNM stage (*P*=0.005), while gender, age and lymph node metastasis had no associations with hsa-miR-429 expression level. High expression level of miR-429 in the tissue sample appears to be associated with low survival in patients with bladder cancer (Figure 1). For patients with low hsa-miR-429 expression, all survived 5 years after surgery, while for those with high hsa-miR-429 expression, only 58% survived. CDKN2B was decreased in bladder cancer compared with matched normal urothelium. Hsa-miR-429 and CDKN2B were inversely expressed in bladder cancer. The data are provided in Table 2.

**Table 1:** Correlation between hsa-miR-429 expression and clinicopathological characteristics of bladder cancer patients

Characteristics	Expression of has-miR-429		P value
	High (n=40)	Low (n=10)	
Gender			
Male	30(78.9%)	8(21.1%)	0.575
Female	10 (83.3%)	2(16.7%)	
Age			
<=60	31(81.5%)	7(18.5%)	0.587
>60	9(75.0%)	3 (25.0%)	
Histological grade*			
PUNLMP/Low-grade	7(46.7%)	8(53.3%)	P<0.001*
High-grade	33(94.2%)	2(5.8%)	
Lymph node metastasis (N)**			
N0	35(79.5%)	9(20.5%)	0.663
N1 or above	5(83.3%)	1 (16.7%)	
TNM stage**			
0/I	10(55.5%)	8(44.5%)	0.005*
II/III/IV	30(93.7%)	2(6.3%)	

*PUNLMP, Papillary Urothelial Neoplasm of Low Malignant Potential; Low-grade, Low-grade Papillary Urothelial Carcinoma; High-grade, High-grade Papillary Urothelial Carcinoma (http://www.pathology.jhu.edu/bladder);

**TNM according to the seventh edition of staging TNM of Union Internationale Contre Le Cancer (UICC) in 2009.

**Table 2:** The expression of hsa-miR-429 and CDKN2B in bladder cancer

	Tumor (n=50)	Normal (n=50)	P value	Pearson's coefficient correlation*	
hsa-miR-429 expression [2^−ΔCt (hsa-miR-429-U6)^]#	0.49±0.07	0.12±0.03	0.019	R=−0.602	P=0.031
CDKN2B expression# [2^−ΔCt (CDKN2B-TBP)^]	0.29±0.08	0.79±0.15	0.014		

#The relative expression levels were evaluated using Real-Time qPCR. Data are shown as mean±SEM.

*log2-Fold-Change (Tumor/Normal) of hsa-miR-429/CDKN2B in 50 bladder cancer patients.

### Hsa-miR-429 decreased the expression of CDKN2B

**Figure 2:**
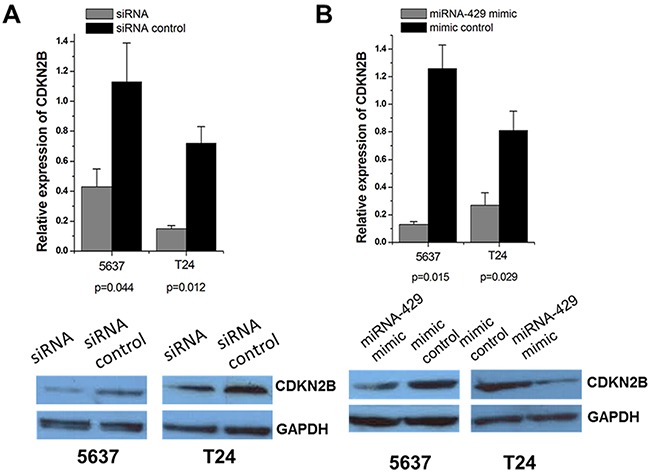
Expression changes of CDKN2B after transfection of CDKN2B siRNA and hsa-miR-429 mimic in T24 and 5637 cells The relative mRNA expression levels were evaluated using Real-Time qPCR. Data are indicated as mean±SD. CDKN2B protein levels were determined using Western blot assay. Each experiment was performed in triplicate for three independent times. **(A)** CDKN2B specific siRNA significantly decreased the expression of CDKN2B at mRNA levels. Representative images of Western blot results indicated CDKN2B specific siRNA significantly decreased the expression of CDKN2B at protein levels. **(B)** Hsa-miR-429 mimic significantly decreased the expression of CDKN2B at mRNA levels. Representative images of Western blot results indicated that hsa-miR-429 mimic significantly decreased the expression of CDKN2B at protein levels.

Forty-eight hours after transfection, CDKN2B expression levels were measured by qRT-PCR and western blot analysis. Both CDKN2B siRNA and hsa-miR-429 mimic decreased CDKN2B at mRNA and protein levels in T24 and 5637 cells (Figure 2A and 2B).

### Hsa-miR-429 reduced the luciferase activities

**Figure 3:**
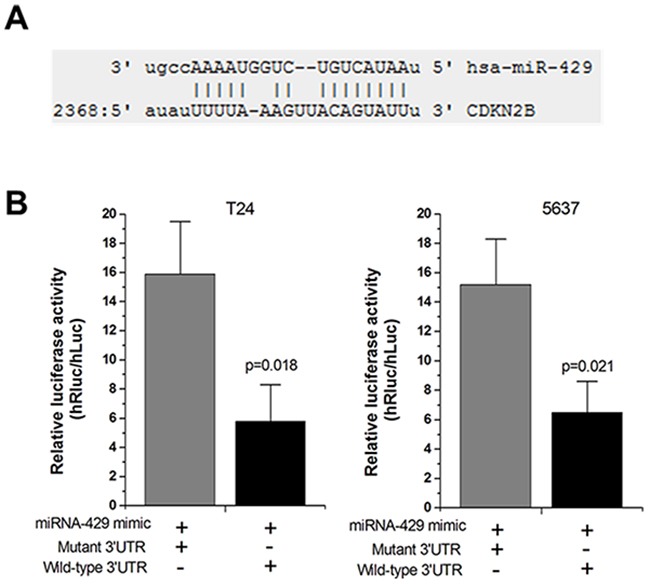
Hsa-miR-429 mimic reduced the luciferase activities **(A)** The predicted hsa-miR-429 binding site located in CDKN2B 3’UTR. **(B)** The relative luciferase activities were inhibited in the cells transfected with the reporter vector CDKN2B 3’UTR-WT, not in the cells transfected with the reporter vector CDKN2B 3’UTR-MUT. Data are shown as mean ± SD. Each experiment was performed in triplicate for three independent times.

Forty-eight hours after transfection, the dual luciferase reporter assay was performed. Hsa-miR-429 inhibited the luciferase activities in T24 and 5637 cells transfected with the reporter vector CDKN2B 3’UTR-WT, but not in cells transfected with the reporter vector CDKN2B 3’UTR-MUT (Figure 3A and 3B), which indicated the hsa-miR-429 binding site within CDKN2B 3’UTR was functional.

### Cell proliferation and apoptosis changes caused by transfection of CDKN2B siRNA/hsa-miR-429 mimic

**Figure 4:**
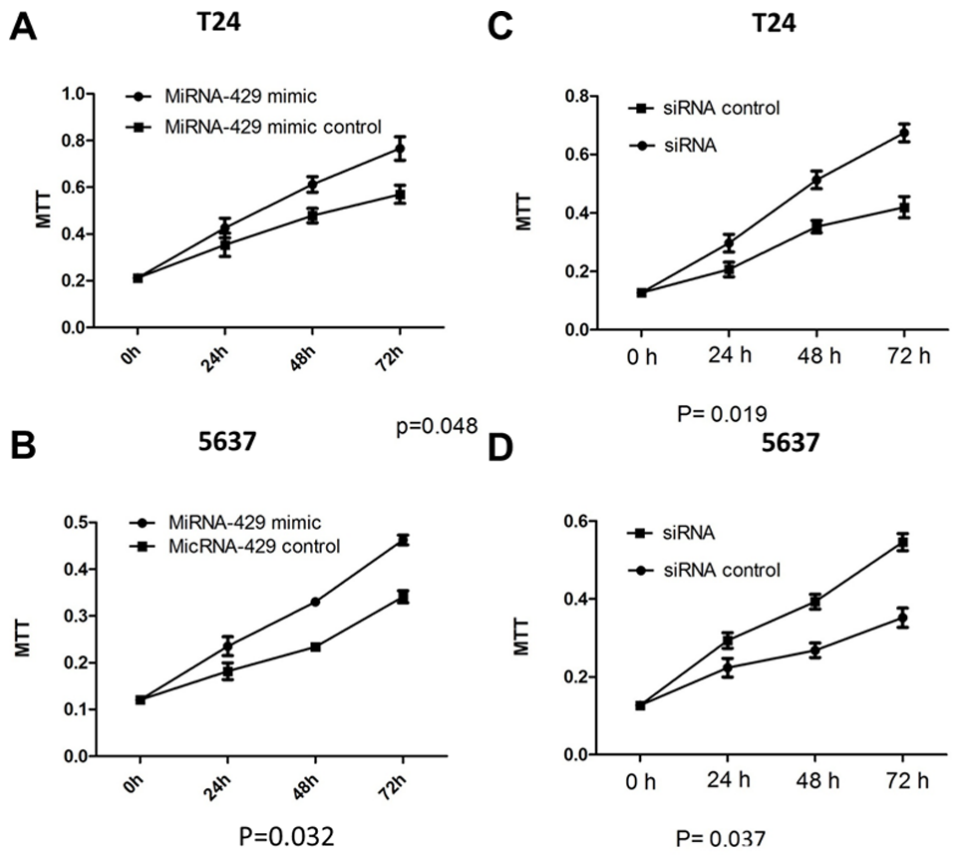
Cell proliferation changes caused by transfection of CDKN2B siRNA and hsa-miR-429 mimic Cell proliferation was measured by MTT assay **(A)** and **(B)** Hsa-miR-429 mimic promoted T24 and 5637 proliferation. **(C)** and **(D)**. CDKN2B siRNA promoted T24 and 5637 proliferation. Data are indicated as mean ± SD. Each experiment in both cell lines was performed in triplicate for three independent times.

**Figure 5:**
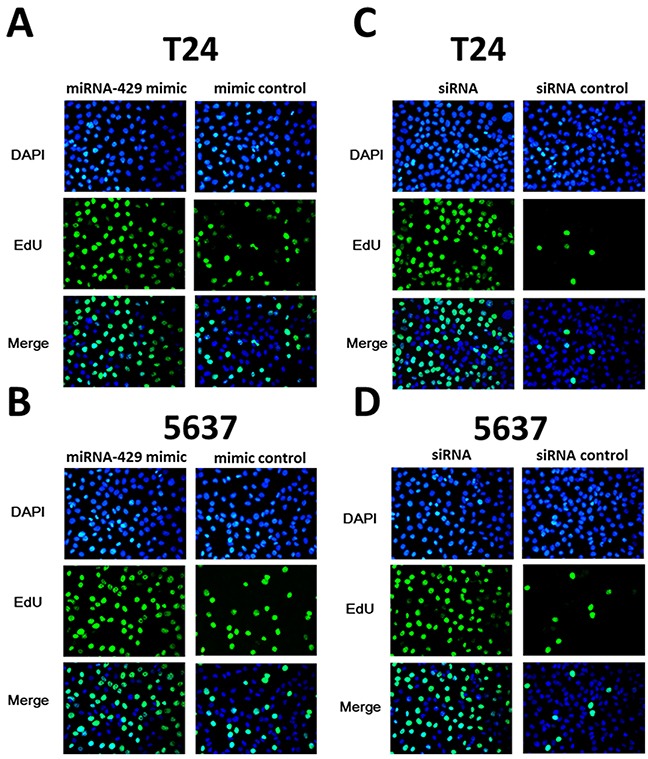
The effects of CDKN2B siRNA /hsa-miR-429 mimic on cell proliferation determined by EdU assay **(A)** and **(B)** Hsa-miR-429 mimic promoted T24 and 5637 proliferation. **(C)** and **(D)**. CDKN2B siRNA promoted T24 and 5637 proliferation.

**Figure 6:**
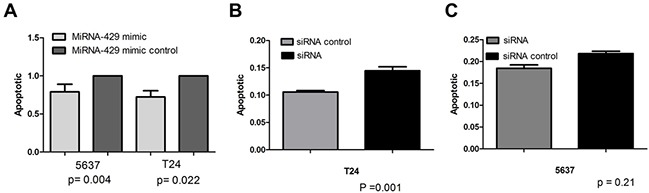
Cell apoptosis changes caused by transfection of CDKN2B siRNA and hsa-miR-429 mimic forty-eight hours after transfection, cell apoptosis changes were determined by Caspase 3 ELISA **(A)** Hsa-miR-429 mimic inhibited T24 and 5637 apoptosis. **(B)** and **(C)**. CDKN2B siRNA inhibited T24 and 5637 apoptosis. Data are indicated as mean ± SD. Each experiment in both cell lines was performed in triplicate for three independent times.

Forty-eight hours after transfection, cell proliferation was determined using MTT assay. Cell proliferation promotion was observed in T24 and 5637 cells by transfection of hsa-miR-429 mimic (Figure 4A and 4B) or CDKN2B siRNA (Figure 4C and 4D). EdU incorporation assay was also used as a further study to determine the effects of CDKN2B siRNA/hsa-miR-429 mimic on proliferation of bladder cancer cell lines. The results showed that CDKN2B siRNA/hsa-miR-429 could promote the cell growth of T24 (Figure 5A and 5B) and 5637 (Figure 5C and 5D) remarkably. As revealed by ELISA assay, decreased cell apoptosis was observed in both bladder cancer cells by transfection of hsa-miR-429 mimic (Figure 6A) or CDKN2B siRNA (Figure 6B and 6C).

### Overexpression of hsa-miR-429 promoted *in vivo* tumor growth

**Figure 7:**
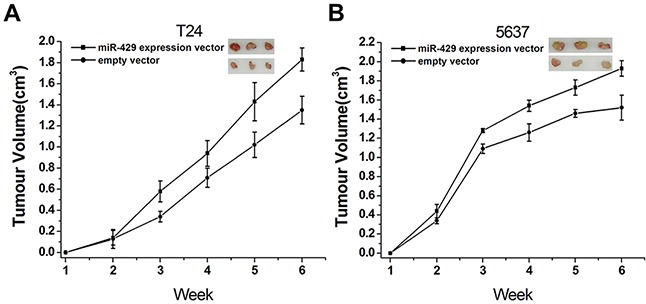
Involvement of hsa-miR-429 *in vivo* cell growth **(A)** Overexpression of miR-429 inhibited T24 cell growth *in vivo* (p < 0. 01). **(B)** Overexpression of miR-429 inhibited 5637 cell growth *in vivo* (p < 0.01). Data are shown as mean ± SD.

To investigate the possible impacts of hsa-miR-429 on *in vivo* growth of bladder cancer cells, the cell growth changes of T24 (Figure 7A) and 5637 (Figure 7B) cells were determined by tumourigenicity assay. Significant differences were demonstrated between the miR-429 expression vector and empty vector-transfected cells. Has-miR-429 was able to promote tumor development, compared to empty control vector treatment. These results further confirmed the positive role of has-miR-429 in the growth of bladder cancer.

## DISCUSSION

In this study, we found that the expression levels of hsa-miR-429 were increased in bladder cancer. Hsa-miR-429 mimic promoted bladder cancer cell proliferation both *in vitro* and *in vivo* and decreased apoptosis. These findings suggested that hsa-miR-429 is an oncogene in bladder cancer.

The data also reveal that the expression levels of CDKN2B were decreased in bladder cancer. Down-regulation of CDKN2B promoted bladder cancer cell proliferation and decreased apoptosis. These effects were similar to that of transfection of hsa-miR-429 mimic in bladder cancer cells.

CDKN2B was an *in silico* predictable target of hsa-miR-429. The mature hsa-miR-429 sequence was partially complementary with CDKN2B 3’UTR target sequence. In consistent with the prediction, we showed that hsa-miR-429 and CDKN2B were inversely expressed in bladder cancer. Hsa-miR-429 mimic decreased the expression of CDKN2B at both mRNA and protein levels. Binding site was confirmed between hsa-miR-429 and CDKN2B. These findings suggested that CDKN2B is a real target of hsa-miR-429. In this study, we identified that overexpression of hsa-miR-429 is a novel contributor to the down-regulation of CDKN2B in bladder cancer, suggesting that post-transcriptional inhibition plays roles in repressing CDKN2B expression.

The exact role of hsa-miR-429 in bladder cancer is still controversial. Hsa-miR-429 is considered to have a tumor suppressive role based on the previous report in which hsa-miR-429 was found to reverse epithelial-mesenchymal transition in bladder cancer. However, in our works, hsa-miR-429 seems to be a promoter of proliferation in bladder cancer cells. Interestingly, it has also been concluded that TGFβ mediates downregulation of hsa-miR-429 [20] and that TGFβ decreases cell viability and induces epithelial-mesenchymal transition in bladder cancer [21]. We speculated that these seemingly contradictory results from hsa-miR-429 may reflect the diversity of TGFβ functions. TGFβ may exert these opposite effects partially through down-regulating hsa-miR-429.

In conclusion, overexpression of hsa-miR-429 contributes to the underexpression of CDKN2B in bladder cancer. Hsa-miR-429 promotes bladder cancer development via down-regulating CDKN2B.

## MATERIALS AND METHODS

### Patient samples

The primary tumor samples and paired adjacent normal urothelia were obtained from 50 patients with bladder urothelial carcinoma newly diagnosed at the Department of Urology, Shenzhen Second People’s Hospital. The clinicopathological features of the patients are provided in Table 1. The informed consent from the patients was obtained. All the patients have been followed up for at least 5 years. All procedures performed in studies involving human participants were in accordance with the ethical standards of the Institutional Review Board of the Second People’s Hospital of Shenzhen and with the 1964 Helsinki declaration and its later amendments or comparable ethical standards.

### Cell culture and transfection

Bladder cancer T24 and 5737 cells and human embryo kidney 293T (HEK 293T, 293T) cells were purchased from the Institute of Cell Research, Chinese Academy of Sciences, Shanghai, China. The cells were routinely cultured in DMEM (Invitrogen, Carlsbad, CA, USA) plus 10% fetal bovine serum.

The cells were transfected with CDKN2B siRNA (target sequence: GAGAGCAATTGTAACGGTTAA)/Allstars Negative Control siRNA (final concentration: 20nM) (Qiagen, Hilden, Germany) or hsa-miR-429 mimic/negative control mimic (final concentration: 100nM) (Ribo, Guangzhou, China) using Nanofectin^TM^ Transfection Reagent (Excell Bio, Shanghai, China).

### Total RNA extraction and reverse transcription

Total RNA was routinely extracted from the tissues or cells using TRIzol reagent (Invitrogen, Carlsbad, CA, USA). Ten micrograms of total RNA was converted to cDNA using the All-in-One^TM^ miRNA qRT-PCR Detection Kit (GeneCopoiea Inc, Rockville, MD, USA).

### Real-time quantitative polymerase chain reaction (qPCR)

Hsa-miR-429 and homo sapiens snRNA U6 qPCR primers were purchased from GeneCopoiea Inc, Rockville, MD, USA. CDKN2B primers were forward:5’-GGAATGCGCGAGGAGAACAA-3’,reverse:5’-CATCA TCATGACCTGGATCGC-3’; TBP primers: 5’-CCCGAA ACGCCGAATATAATCC-3’ (forward), 5’-AATCAGTG CCGTGGTTCGTG-3’(reverse). The PCR procedures were as previously described [22, 23]. Expression fold changes were calculated using 2^−ΔΔCt^ methods [24].

### Western blot assay

Western blotting was performed as described elsewhere [25]. Antibodies specific to CDKN2B and GAPDH were obtained from Santa Cruz, Dallas, Texas, USA.

### miRNA target prediction

miRanda (http://www.microrna.org), DIANA - microT v3.0 (http://www.http://diana.cslab.ece.ntua.gr/microT) and TargetScan (http://www.Targetscan.org) were used to predict binding sites between hsa-miR-429 and CDKN2B 3’UTR.

### Dual luciferase reporter assay

The reporter vector CDKN2B 3’UTR-wild-type (WT) was created by chemically synthesizing and cloning the CDKN2B 3’UTR fragment 5’-ATATTTTTAAAGTTA CAGTATTT-3’(position2368, NM_004936) containing the predicted hsa-miR-429 binding site into the Xhol and Notl sites of the psiCHECK-2 vector (Promega, Madison, WI, USA). The reporter vector CDKN2B 3’UTR-mutated-type (MUT) was created by mutating the hsa-miR-429 seed region binding site (seed sequence binding fragment 5’-AGTATT-3’ changed to 5’-CCAGAA-3’).

### Cell proliferation and cell apoptosis assays

Cell proliferation was determined using MTT assay. Apoptosis caused by CDKN2B siRNA and hsa-miR-429 mimic was determined using the Caspase 3 ELISA assay kit (R&D, Minneapolis, MN, USA). The procedures for cell proliferation and cell apoptosis assays were performed as previously described [22].

### EdU incorporation

Cell proliferation was also detected using the EdU incorporation assay (Beyotime Inst Biotech, China). Briefly, 1×10^5^ cells were seeded in each well of a 96-well flat-bottom plate. The cells within a single well were incubated with 100 μl of 50 μM EdU for 2 h, and then fixed with 50μl of fixing buffer for 30 min at room temperature. After removing the buffer, the cells were incubated with 50μl of 2 mg/ml glycine for 5 min and then washed with 100μl of PBS. The cells were also added with 100μl of permeabilization buffer followed by washing with 100μl of PBS. Subsequently, cells were added with 100μl of 1X Apollo solution for 30 min at room temperature in the dark. After that, cells were incubated with 100μl of 1X DAPI solution for 30 min at room temperature in the dark followed by washing with 100μl of PBS. The cells were finally observed by fluorescence microscopy.

### Tumourigenicity assay

All experiments involving animals were approved by Institutional Review Board of Shenzhen 2^nd^ People’s Hospital (Shenzhen, China). Stable transfection cell lines were constructed in our lab by transfecting the miR-429 expression vector or empty vector into T24 and 5637 cells and selecting with G-418. Four-week-old BALB/c athymic nude mice were bred in laminar flow cabinets and cultured at a constant humidity and temperature (25–28°C). In detail, 1×10^7^ T24 or 5637cells were suspended in PBS and injected subcutaneously into the flank of nude mice (3 in each group) on day 0. Tumor growth was monitored by the tumor volume, which was calculated by the formula: Volume (mm^3^) = width^2^ (mm^2^) × length (mm) /2. The mice were sacrificed on day 42, and the tumors were excised. The *in vivo* experiment was repeated three times.

### Statistical analysis

Paired samples t-test was used to analyze the expression differences between bladder cancer and matched urothelium. Pearson’s coefficient correlation was used for expression correlation assay. Survival curves were plotted using the Kaplan–Meier method and compared using the log-rank test. Independent samples t-test was used to analyze expression changes after transfection, luciferase activity and cell apoptosis. ANOVA was used to analyze cell proliferation. SPSS (Version 19.0) was used for statistical analyses. All P values were two-sided. A P value <0.05 was considered to be statistically significant.
